# The physical demands of the match according to playing positions in a South African Premier Soccer League team

**DOI:** 10.17159/2078-516X/2024/v36i1a16752

**Published:** 2024-07-15

**Authors:** M Rhini, R C Hickner, R Naidoo, T Sookan

**Affiliations:** 1 Discipline of Biokinetics, Exercise and Leisure Sciences, School of Health Sciences, University of KwaZulu-Natal, South Africa; 2 Department of Nutrition and Integrative Physiology, Institute of Sports Sciences and Medicine, College of Health and Human Performance, Florida State University

**Keywords:** physical demands, formation, playing position, total distance

## Abstract

**Background:**

Evidence indicates that international soccer players cover a total distance of between eight and 14 km in a match. Approximately 80 – 90% of this distance constitutes low-intensity actions, and about 10 – 20% is high-intensity. These data are influenced by playing position, formation, league standard, and national differences in different countries.

**Objective:**

To quantify the physical demands of competitive matches on the different playing positions in a South African Premier Soccer League team.

**Methods:**

A descriptive study design included 21 players on the same team. Data were collected over 23 official matches during the 2019/2020 season using PlayerTek GPS devices (10Hz). The data collected included total distance covered, high-intensity running distance, power plays, top-end speed, and distance per minute.

**Results:**

Statistical differences were evident only in the high-intensity running distance and power plays. The centre-forwards (p<0.001), attacking central midfielders (p=0.006), and full-backs (p=0.01) covered the most high-intensity running distance (p<0.001) than centre-backs. The attacking central midfielders and centre-forwards recorded more power plays than the centre-backs. Total distance (p=0.01), power plays (p=0.004) and distance per minute (p=0.001) were lower in the second half than in the first half of the match.

**Conclusion:**

Centre-forwards performed more high-intensity actions, whereas the centre-backs sprinted less than all the other positions. These data provide insight into the positional differences that exist and could help coaches to prescribe position-specific training programmes.

The available literature indicates that international outfield soccer players record a total distance between eight and 14 km in an official match. ^[1,2]^ Of this total distance, 10 to 20% involves high-intensity actions, which include explosive movements such as sprints, jumps, accelerations, and decelerations. It is reported that players sprint between 700 and 3900 m in high-intensity running (19.8 – 25.1 km·h^−1^), and between 200 and 600 m in very high-intensity running (above 25.1 km·h^−1^), depending on playing position. ^[2]^ According to the literature, these physical demands are influenced by several contextual and situational factors, including tactics, formations, the quality of the opposition, the score line, the standard of the league, and the cultural differences in playing style in various countries. ^[3,4]^

Data from European soccer indicate that centre-backs cover the least total distance of all outfield players, ^[2]^ generally between nine and 10 km in a match. Central midfielders and wide midfielders generally cover the most total distance, from 11 to 14 km, depending mainly on the tactical formation and style of play. ^[5,6]^ The total distance covered by the full-backs and centre-forwards varies across the literature. For example, some studies suggest that the full-back position is one of the most physically demanding positions ^[2,7]^, while others report that full-backs cover a distance between nine and 10 km in a match, which is relatively less than other positions. ^[1]^ One of the reasons for this is that a full-back in a 1-3-5-2 formation is more likely to cover greater distances than a full-back in a 1-4-4-2 formation due to the number of players occupying the wide space. ^[4]^ Similarly, some studies have indicated that centre-forwards could cover between 11 and 12 km, ^[2,6]^ while others indicate between 9 and 10 km in an official match. ^[7,8]^ This is also down to the tactical demands imposed by the different tactical formations.

Similarly, the interactive effect of the contextual factors is well documented. For example, a study on Brazilian professional soccer showed that the total distance covered in the lower divisions is less than the total distance covered by teams in the premier division. ^[3]^ However, in England, in the lower divisions, such as the English Championship and League One, it is reported that players tend to record higher total distance compared to the English Premier League (EPL) players. ^[1]^ In South Africa, the available literature indicates that the central midfielders and attacking central midfielders cover the longest total distance – between eight and nine km – while the centre-backs and centre-forwards cover the shortest total distance, of between seven and eight km in an official match. ^[9]^ In comparison with European data, the physical demands in South Africa are relatively less across playing positions. The variations in this data confirm that indeed contextual factors play a significant role in the physical demands of the match. Thus, it is important to consider these factors when quantifying the physical demands, as they have direct implications for the periodisation of training. ^[8]^

The physical match data helps coaches design tailor-made training programmes based on the demands of the game. It is the physical and physiological demands of the game, as per the positional differences and style of play, that determine the training load that players should be exposed to in order to optimise competitive performance. Moreover, it is reported that inappropriate training loads (over-loading or under-loading) are associated with an increased risk of injuries, fatigue and poor performance. ^[8]^ Therefore, it is important that the game’s physical demands are accurately quantified across the world and contextualized in the relevant situational and environmental factors to prevent any training load error. To the best of the researchers’ knowledge, there is limited literature on physical match performance in the South African Premier Soccer League. Therefore, the aim of this study was to quantify the physical match performance of South African professional soccer players in official matches, according to positions.

## Methods

### Match sample

The study used a descriptive, observational research design, where data were collected from a single Premier Soccer League (PSL) team over 27 weeks during the 2019/2020 season. A total of 23 (339=files)) official matches (league and tournament matches) were included in the study. The team participated in the PSL, the top professional division in South Africa, comprising 16 teams. Twenty-one players (25.8 ± 3.8 years of age; 170.0 ± 6.0 cm height; 72.2 ± 9.9 kg body weight; and 23.1 ± 2.2 kg/m^2^ body mass index) participated in the study. This included six (6) different playing positions, namely: goalkeepers (n = 7); full backs (n = 61); centre-backs (n = 55); central midfielders (n = 94); attacking central midfielders (n = 55); and centre-forwards (n = 67). Only players who played for the entire 90 minutes were included in the study, and goalkeepers were excluded from the overall analysis, therefore the sample consisted of 332 files (n). The team played in a 1-3-4-2-1 formation when attacking, and 1-5-4-1 when defending in all matches.

### Ethical considerations

Permission to conduct the study was sought from the team’s management and the coaching staff. The University of KwaZulu-Natal’s Biomedical Research Ethics Committee granted ethical clearance (BE695/18). Before entering the study, all players were informed of the nature and demands of the study and the potential risks. Furthermore, all players gave written, informed consent in accordance with the Helsinki Declaration.

### Data collection

Data were collected using a GPS device (PlayerTek, Dundalk, Ireland), and all players were required to wear the devices secured in a pocket in the vest provided by the company. The pocket in the vest was located at the back, between the upper scapulae, at approximately the T3–4 junction of each player. All devices were turned on 15 minutes prior to the warm-up to allow acquisition of satellite signals, and they were taken off the players and returned at the end of every match. A trial period was allocated for all the players to familiarise themselves with the device. The GPS device had a sampling rate of 10Hz and an accelerometer sampling rate of 100Hz. The accuracy and reliability of the PlayerTek units has been documented in a previous study. ^[10]^ The unit provided information on total distance (km); high-intensity running (speed above 18 km·h^−1^ in m); ^[11]^ power plays, defined as significant actions such as acceleration or high-speed running in which power output is above 20 watts per kilogram of body weight; distance per minute (m·min^−1^) and top end-speed (km·h^−1^).

### Statistical analysis

All data analysis was conducted using the statistical software SPSS, version 25 (IBM, Chicago, USA). Descriptive statistics were presented as the mean ± standard deviation (SD). To determine the differences between positions, analysis of variance (ANOVA) was used. When the significant difference was found, a Tukey’s post hoc test was performed to assess where the difference occurred. A student’s paired t-test was performed to compare the first half versus the second half of the match. To compare the influence of situational factors on performance variables, a student’s paired t-test was also used to test the influence of match location (home/away), with Cohen’s test (*d*) used to calculate effect sizes. In order to test the influence of the match outcome (win, draw or lose), repeated measures ANOVA was used, with the partial eta squared (*n*^2^) effect sizes calculated to test the magnitude of the difference. Cohen’s d effect sizes were interpreted as follows: small (=0.2); medium (=0.5); or large (=0.8), and the partial eta squared were interpreted as: small (>0.01); medium (>0.06); or large (>0.14). The level of significance was set at p<0.05 for all analyses.

## Results

The descriptive statistics for the players are presented below, in Table 1. Results from ANOVA showed that there was no statistical difference in the total distance across playing positions F (4, 15) 1.83, p = 0.17). In contrast, the results show that high-intensity running differs significantly from position to position, F (4, 15) = 8.19, p = 0.001). According to the Tukey’s post hoc test, the centre-forwards (1504 ± 202 m) covered significantly more high-intensity running than centre-backs (p = 0.001) and central midfielders (p = 0.03). The attacking central midfielders (1432 ± 188 m, p = 0.006) and full backs (1309 ± 150 m, p = 0.01) also covered more high-intensity running than centre-backs, although not significantly higher than the central midfielders. The centre-backs (735 ± 137 m) recorded the lowest values than the centre-forwards, attacking central midfielders and full backs. Similarly, analysis of power plays showed that attacking central midfielders recorded more power plays than centre-backs F (4, 15) = 4.23, p = 0.01); and centre-forwards recorded higher values than centre-backs (p = 0.04). However, no significant difference was found in top-end speed F (4, 15) = 0.29, p = 0.87) and distance per minute F (4, 15) = 2.45, p = 0.09) across all field positions.

The analysis comparing (Table 2) the first half and the second half of matches showed a significant decline in overall total distance covered (p = 0.001), power plays (p = 0.004), and distance per minute (p = 0.001). However, there was no significant change in high-intensity running and top-end speed across all field positions.

Table 3. shows the means ± SD and effect sizes for the influence of the situational and environmental variables (match location and match outcome) on the demands of the game. The match outcome data were analysed using repeated measures ANOVA. This found no significant difference in total distance covered, power plays or high-intensity running (p = 0.005). A significant difference was only evident in the average top-end speed and distance per minute, depending on the match outcome F (2, 20) = 72.11, p = 0.005). The post hoc analysis showed that the average top-end speed for drawn matches (M=29.8) and for won matches (M=28.0) significantly exceeded the average top-end speed for lost matches (M=17.7), p<0.005 in both cases; whereas the post hoc analysis of distance per minute, showed that the values were significantly higher during won (98.1 ± 5.9 m·min^−1^, p = 0.03) and drawn matches (98.8 ± 6.2 m·min^−1^, p = 0.03) compared to lost matches (94.6 ± 6.0 m·min^−1^). However, when analysing the effect sizes, large effect sizes (*n*^2^) were found in all variables (total distance covered = 0.24; high-intensity running 0.37; power plays = 0.38; top-end speed = 0.88; and distance/minute = 0.47) across all three different match outcomes. Effect sizes were also analysed for match location (home/away), and small effect sizes (*d*) were found in all variables.

## Discussion

The aim of this study was to measure the physical demands placed on players in different positions during competitive soccer matches in the South African Premier Soccer League. A statistically significant difference was evident in only two dependent variables (high-intensity running and power plays) across outfield playing positions. There was no statistical difference in total distance covered between playing positions, which contradicts with the majority of previous studies, which have reported positional differences in total distance covered.^[4,5,8]^ Although there was no difference in total distance covered between playing positions, the results showed that, overall, the outfield players covered a mean total distance of 10.2 ± 0.7 km, which is comparable with other studies from Italian Seria A, Portuguese LigaPro and Brazilian professional divisions. ^[2,3,6]^

The analysis of the high-intensity running data showed that the centre-forwards performed the most high-intensity running, followed by attacking central midfielders and full backs, compared to the rest of the positions. This agrees with findings from a study by Baptista et al.^[12]^ where they reported that the centre forward position is the most physically demanding, with the players performing more high-intensity running than other positions. Although the authors did not indicate the possible reason for this, in the present study, this could be due to the tactical formation used by the team. The team used a 1-3-4-2-1 tactical formation when in possession, and a 1-5-4-1 when out of possession throughout the season. In this formation, it is expected that the centre forward would press high when not in possession, and would likely be an outlet or exit point when in possession, especially during break and counter-attack moments. These tactical demands tend to place greater physical demands on the centre forward. In support of this, findings from a study conducted by Modric et al.,^[4]^ where they investigated the running performance of UEFA Champions League players, showed that the centre-forwards covered the most total distance, with the second-most high-intensity running, compared to all the other positions. Moreover, they indicated that these distances were covered during out-of-possession moments. Ju et al.^[13]^ went further and reported that, in the EPL, the centre-forwards were involved in more ‘out-of-possession’ moments compared to any other position. The authors attributed this to the tactical trends, such as ‘pressing’ tactics and the use of tactical formations (e.g. 1-5-4-1; 1-4-5-1) which use a single centre forward, as in the present study. This data reflects the amount of work centre-forwards perform without the ball, which is not always noticeable. Furthermore, it illustrates that the centre-forwards play a vitally important tactical role, not only when in possession, but more so when out-of-possession. Hence, coaches should be aware of this data when designing training programmes.

The attacking central midfielders performed the second-most high-intensity running. In the present study they were generally wide midfielders who inverted into the advanced central space in order to create space for the full backs to overlap when in possession. They were expected to progress the ball, and to provide immediate support to the lone centre forward, especially during break- and counter-attack moments. However, when out-of-possession they were more likely to move to the wide zone to close down/press the opposition’s full backs. In a situation where the press was unsuccessful, they were expected to drop back to form the second defensive block with the central midfielders. Typically, this increased the demands placed on the attacking central midfielders, which is reflected in their high-intensity running data. This is also confirmed by Ju et al. ^[13]^ who showed that the central offensive players performed more high-intensity running when ‘closing down/pressing’, ‘running in behind’ and during ‘support play’ during peak moments than any other position.

The full-backs in the present study were expected to cover the entire wide space, both when in possession and out-of-possession; hence, they recorded the third-most high-intensity running, which corroborates the available literature. ^[14]^ Modric et al. ^[15]^ analysed the influence of different formations on physical performance in the game, and they found that full backs performed more high-intensity running in the 1-3-5-2 formation than in other formations. This is due to the way teams set up in a ‘three-at-the-back’ formation, with the full back assuming the role of wing backs, as the only players expected to cover the entire wide area. According to the literature, the players in the wide spaces generally perform significantly more high-intensity running compared to players in the central space. ^[11]^ This could explain why the central midfielders and centre-backs performed the least high-intensity running in this study, which is also consistent with most of the available literature. ^[1,3]^ According to the literature, the centre back position, in particular, seems to be the least demanding position, regardless of formation and tactics. ^[16]^ Vilamitjana et al. ^[5]^ investigated the high-intensity actions for playing positions with different formations (1-3-4-3 and 1-4-2-1-3), and they observed that the centre-backs performed the least high-intensity running and the fewest sprints, and had the lowest mean heart rate of all positions, regardless of tactical formation. However, when comparing the centre-backs data in different formations, evidence showed that centre-backs in a ’three-at-the-back’ formations perform more high-intensity running than centre-backs in a ‘four-at-the-back’ formation. Thus, it is warranted that coaches are aware of the influence of different formations on the physical demands of the centre back position.

Concerning the power plays, the attacking central midfielders performed the most power plays, followed by the centre-forwards and the full backs; while the central midfielders and centre back recorded the least. By definition, power plays constitute a significant action, such as acceleration or high speed running, in which power output is above 20 watts per kilogram of body weight. However, currently there is no comparable data available for power plays in soccer. No significant difference was found in top end-speed across positions. This could indicate that players are exposed to the same speed demands: whether in the wide or central space, any player can reach different maximum speeds depending on their physical capability. Although this may be the case in the present study, it should be noted that the available literature suggests that players in the central space (centre back and central midfielders, excluding the centre-forward), generally have lower top end-speeds compared to players in the wide spaces (full-backs and wide midfielders). ^[7]^ The rationale for this could be that wide players are exposed to greater opportunities to perform longer sprints, compared to players in the central space who do a lot of short and sub-maximal sprints.^[11]^ It is reported from previous research that the centre-forwards tend to reach the highest top end-speed in the match, more than any other position, possibly due to the fact that speed and quickness are some of the key qualities that a centre-forward needs to be successful. ^[7]^ In addition, the centre-forward position is one position where a player has the freedom to manoeuvre across the central and wide zones, depending on the tactics and formation employed. In relation to the interactive effect of other contextual factors, the relative contributions of match location, and the results of the match were measured. The analysis of the results showed that there was no significant difference in the total distance covered, power plays and high-intensity running performed, whether at home, or away. Although these findings were not significant, it is interesting that the influence of home advantage was not so evident. Previous studies have indicated that teams are more likely to produce more high-intensity actions when playing at home than when playing away. ^[2,3]^ Gonçalves et al. ^[14]^ also reported the influence of home advantage on physical performance, where they demonstrated that home fixtures tend to result in more high-intensity running compared to away fixtures. This can be attributed to a wide range of factors, including the tactical formation and style of play that the coaches employ when playing home matches, which would normally be proactive and offensive, rather than defensive. Another factor could be the crowd effect, no ill-effects from travelling, familiarity with the home-ground environment and overall psychological factors, which give players a feeling of confidence and a more positive physiological outlook. ^[17]^ In the current study it is not clear why the influence of home advantage is not evident. However, it is possibly because the reference team is a relatively small, mid-table team that does not have a large fan base. As a result, it is likely that wherever the team played, the psychological aspects and the impact of the fans had less or no effect on the performance.

No significant difference was evident across the variables (with the exception of top end-speed and distance per minute) when analysing the influence of the outcome of the match. However, large effect sizes were observed in variables across all three match outcomes. In this case, a large effect size indicates that a practical significance exists even though there is no statistical difference. Hence, when the match ended in a draw, the average total distance covered was 9.8 km, with 77 power plays, and high-intensity running of 1129 m. When the match ended in a win, total distance covered was 9.5 km, there were 71.7 power plays, and 1032 m of high-intensity running. When the team was losing, total distance covered was 9.5 km, with 69.2 power plays, and 996 m of high-intensity running. These results are partially in agreement with those in the existing literature, which indicate that losing a match is associated with less high-intensity activity. ^[2,14]^ It is reported that losing teams would normally prefer to have control of the game through keeping the ball and playing a patient possession-based style of play, which results in less total distance covered and less intensity. ^[3]^ Whereas, leading teams tend to be more organised defensively (low or medium block), which then decreases their possession and forces them to rely on counter-attacking. ^[18]^ This therefore results into players performing more high-intensity running than plyers in the losing team. This was supported by Aquino et al. ^[19]^ who reported that, when teams won or drew matches, they produced more high-intensity activity than when they lost. In addition, they showed that, when teams are winning, they tend to adopt a more direct style of play with long and fast passes which result in greater physical demands. This could also explain why in the present study the distance per minute was higher when the team won or drew compared to when the team lost.

When comparing the first and second halves, the data in the current study showed that there were only significant differences in total distance covered, power plays and distance/minute, with no significant change observed in top-end speed and high-intensity running. Total distance declined by 9.8% in the second half, and power plays and distance/minute declined by 10.6% and 7.8%, respectively. This decrease in performance can be attributed to underlying factors, such as fatigue or game management tactics.[20] This study was unable to consider the ‘during the game’ score-line and its effects on performance. Moreover, we did not investigate the stage in the second half when the decrease in physical performance became evident.

### Limitations

The current study has some limitations. First, we were unable to analyse the tactical behaviour of the team during the game to give context to the physical performance, especially in situations where a style of play or formation was changed during the match, and when players had changed positions. Any change in the style of play or formation during the match has a direct influence on the physical demands. Secondly, we were not able to consider which team scored first in the match, and how that affected the game. This also determines if a team would be more likely to perform more high-intensity actions, or not. Thirdly, the quality of the opposition and the tactical formations used by the opposition were not considered in this study. A team is more likely to cover greater total distance when playing against a high-quality opposition than against a weaker team. Hence, this should have been considered. Finally, the sample in the study was small; a larger sample size with more players in each position would have been ideal. The reference team used in this study was a mid-table team: a larger sample, with teams from the top four teams (high quality teams) and the bottom of the table (weaker teams) might have yielded more comprehensive findings.

## Conclusion

The results of this study confirm that physical demands vary according to tactical positions, and are a direct consequence of multiple factors: some are controllable (e.g. formation and tactics), while some are not so controllable (match results, quality of the opposition and match location). However, it is evident that some positions, such as centre back, would most likely remain the least physically demanding, regardless of tactical formations. The centre-forward position proved to be the most demanding position, followed by the attacking central midfielders and full backs, particularly when looking at the high-intensity actions (high-intensity running and number of power plays). These findings could help coaches to plan and control training load according to the demands of specific positions. This would ensure that the training load replicates the demands that players are exposed to during the match, which is what will contribute to optimal adaptation.

## Figures and Tables

**Table 1:** Means ± SD for the physical demands of the match, according to playing positions (n=332)

Physical demands	Playing positions					p value: post hoc

	Full-backs n = 61	Centre-backs n = 55	Central-midfielders n = 94	Attacking central-midfielders n = 55	Centre-forwards n = 67	
Total distance (km)	10.4 ± 0.6	9.4 ± 0.1	10.3 ± 0.9	10.9 ± 1.2	9.8 ± 0.7	0.17
HIR (m)	1309 ± 150^b^	735 ± 137	1090 ± 265	1432 ± 188^b^	1504 ± 202^b^^c^	< 0.001: CF, ACM, FB > CB; CF > CM
Number of f PP	88 ± 3	60 ± 10	80 ± 14	100 ± 21^b^	91 ± 14b	0.01: ACM > CB ; CF > CB
TES (km.h^−1^)	26.1 ± 2.8	26.6 ± 2.8	25.2 ± 3.4	28.8 ± 3.4	24.7 ± 9.3	0.87
D/min (m.min^−1^)	104.4 ± 5.1	93.1 ± 1.2	104.7 ± 11.1	113.0 ± 10.4	98.1 ± 1.2	0.91

Data are presented as means ± SD. Differences (p<0.05) between positions indicated using letters (a, b, c, d & e) as follows:

a denotes greater than FB;

b greater than CB;

c greater than CM;

d greater than ACM;

e greater than CF.

HIR, high-intensity running; PP, power plays; TES, top end-speed; D/min, distance per minute. FB, full backs; CB, centre-backs; CM, central midfielders; ACM, attacking central midfielders; CF, centre-forwards

**Table 2:** Means ± SD for first and second half comparison, in overall variables

Physical demands	First half	Second half	95% Confidence Interval of the difference		t value	p value

			Lower	Upper		

Total distance (km)	5.1 ± 0.8	4.6 ± 0.8	0.1	0.7	2.8	0.01*
HIR (m)	605 ± 214	563 ± 195	−1.9	87.7	1.9	0.05
Number of PP	42 ± 3	38 ± 12	1.6	7.4	3.2	0.004*
TES (km.h^−1^)	29.5 ± 2.2	29.4 ± 2.0	−0.4	0.7	0.6	0.52
D/min (m.min^−1^)	105.1 ± 16.9	96.8 ± 16.0	4.7	11.9	4.7	0.001*

Data are presented as means ± SD.

* indicates significant difference (p<0.05).

HIR, high-intensity running; PP, power plays; TES, top end-speed; D/min, distance per minute

**Table 3:** Results and effect size from the analysis of the influence of match location (n= 23) and match outcome (n=23) on physical performance in the match

	Match location			Match outcome			

Physical demands	Home n=12	Away n=11	Effect Size (Cohen’s *d*)	Won n=8	Draw n=7	Lost n=8	Effect Size (partial η^2^)

Total distance (km)	9.8 ± 1.7	9.7 ± 1.7	0.06	9.5 ± 1.8	9.8 ± 2.1	9.5 ± 1.9	0.24
HIR (m)	1143 ± 439	1126 ± 451	0.04	1032 ± 451	1129 ± 489	996 ± 410	0.37
Number of PP	78 ± 27	77 ± 27	0.05	72 ± 27	77 ± 30	69 ± 25	0.38
TES (km.h^−1^)	25.4 ± 3.7	26.0 ± 3.6	0.16	28.0 ± 3.1	29.8 ± 3.3	17.7 ± 2.1*	0.88
D/min (m.min^−1^)	99.4 ± 17.8	97.8 ± 17.7	0.09	98.1 ± 5.9	98.8 ± 6.2	94.6 ± 6.0*	0.47

Data are presented as means ± SD for match location and outcome of the match.

* indicates the significance difference in variables (p<0.05).

Cohen’s d effect sizes were interpreted as follows: 0.2 = small; 0.5 = medium; or 0.8 = large, and the partial eta squared effect sizes (η^2^) were interpreted as: 0.01 = small; 0.06 = medium; and 0.14 = large. HIR, high-intensity running; PP, power plays; TES, top end-speed; D/min, distance per minute
